# A Case of Granuloma Annulare Mimicking Candidal Intertrigo in the Groin

**DOI:** 10.7759/cureus.94157

**Published:** 2025-10-08

**Authors:** Scotty Smith, Patrick Perche, Leonard Gately

**Affiliations:** 1 Department of Dermatology, Louisiana State University Health Sciences Center, New Orleans, USA

**Keywords:** annular lesion, autoimmune skin disease, granuloma annulare, intertrigo mimicker, necrobiotic disorders

## Abstract

Granuloma annulare (GA) is a benign, non-infectious granulomatous skin condition of uncertain etiology, often presenting as annular erythematous plaques. While commonly localized to the hands and feet, GA has multiple clinical variants and can mimic other dermatologic conditions, leading to diagnostic challenges. We report a rare case of GA localized to the inguinal region in a 55-year-old female initially treated empirically for candidal intertrigo without improvement. Biopsy demonstrated interstitial histiocytic infiltrates dissecting between collagen bundles with increased dermal mucin, confirming a diagnosis of interstitial GA. The patient was subsequently treated with triamcinolone 0.1% ointment and hydroxychloroquine 200 mg twice daily and is currently pending follow-up. While GA often resolves spontaneously, refractory cases require individualized management, as no gold-standard therapy exists. The objective of this report is to highlight an atypical presentation of GA and emphasize the importance of maintaining a broad differential diagnosis for annular skin lesions. Further research is needed to elucidate GA’s pathogenesis and optimize treatment strategies for chronic or widespread disease.

## Introduction

Granuloma annulare (GA) is a benign, noninfectious granulomatous dermatosis of unclear etiology that typically presents as annular erythematous plaques, usually asymptomatic but sometimes painful or pruritic [1-5].

Localized GA, the most common variant, is usually self-limiting and affects the hands and feet, whereas generalized GA typically involves the trunk, arms, and legs, and tends to be more chronic and less responsive to treatment [1-5]. Less frequent subcutaneous, patch-type, perforating, and acral variants have also been described [2]. GA predominantly affects females, with a female-to-male ratio of 3:1 and peak incidence in the fifth decade of life [1]. A 2021 cross-sectional study by Barbieri et al. estimated incidence and prevalence in the U.S. at 0.04% and 0.06%, respectively. GA has been associated with several systemic conditions, including diabetes mellitus (DM), hyperlipidemia, and autoimmune diseases such as hypothyroidism, rheumatoid arthritis, and systemic lupus erythematosus [2,4-6]. GA has also been associated with certain malignancies and viral infections such as HIV, Epstein-Barr virus, and Hepatitis B virus [5].

Histopathologically, GA primarily demonstrates either palisading or interstitial granulomatous patterns, with dermal mucin deposition serving as a key diagnostic feature [2,5]. Recent studies suggest a role for cell-mediated inflammation and cytokine dysregulation, including JAK-STAT signaling. This idea has led to successful off-label use of JAK inhibitors as a therapeutic option for GA, with reports of rapid lesion clearance and favorable tolerability; however, further studies are needed [2,3,5,7].

Although GA most often occurs on acral sites, involvement of intertriginous regions is rare and may be misdiagnosed as candidal intertrigo, tinea cruris, or other infectious dermatoses [4,5]. This diagnostic overlap is clinically important as it can delay biopsy and appropriate management. We report a unique case of GA localized to the inguinal folds, emphasizing the need to consider GA in the differential diagnosis of refractory annular lesions in atypical anatomic sites.

## Case presentation

A 55-year-old female with a past medical history of hyperlipidemia (total cholesterol 234 mg/dL), hypothyroidism, multiple sclerosis, and seborrheic dermatitis presented to the dermatology clinic for a persistent rash in the bilateral groin for approximately nine months. The patient described the rash as burning and intermittently pruritic, and she noted no improvement after trying several over-the-counter antifungal creams. Due to the rash’s persistent nature and location, it was significantly affecting her quality of life.

**Figure 1:**
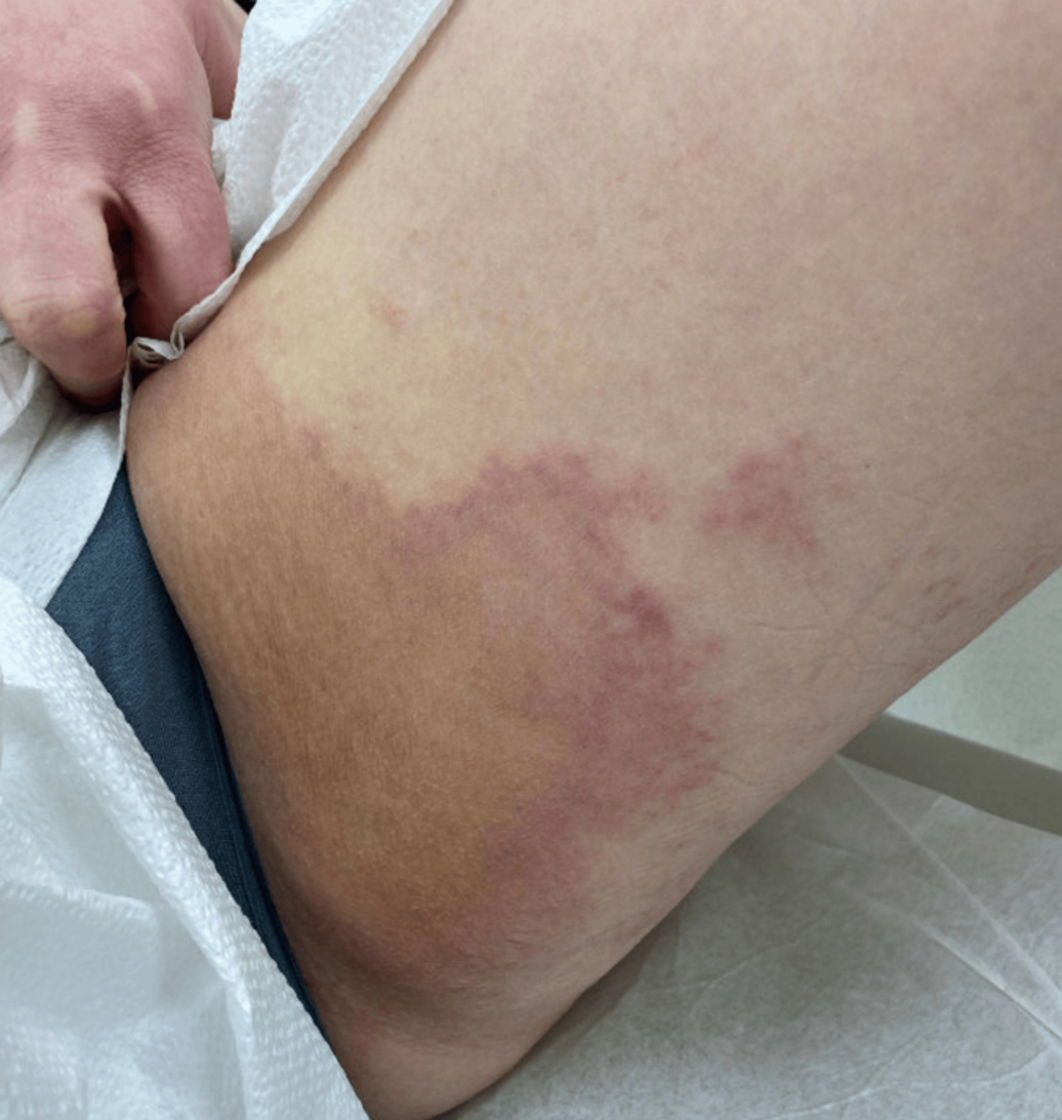
Large skin-colored to yellow, thin annular plaque with erythematous borders with adjacent erythematous papules localized to the left inguinal crease and extending to the proximal medial thigh

Initial physical examination revealed large skin-colored to slightly yellow, thin, annular plaques with erythematous borders and few adjacent coalescing erythematous papules located on the bilateral inguinal creases and extending onto the proximal, medial thighs. No scale was appreciated. Figure 1 reveals the initial presenting rash of the left inguinal region and proximal thigh. Given the location of the rash and the presence of satellite papules, candidal intertrigo was initially suspected, and treatment with fluconazole 200 mg once weekly for three doses was initiated. A systemic antifungal was selected based on patient preference. KOH and fungal culture were not obtained at initial presentation. No improvement was noted after oral fluconazole. She was subsequently prescribed ketoconazole 2% cream and hydrocortisone 2.5% cream to use twice daily in addition to terbinafine 250 mg daily for 21 days.

**Figure 2:**
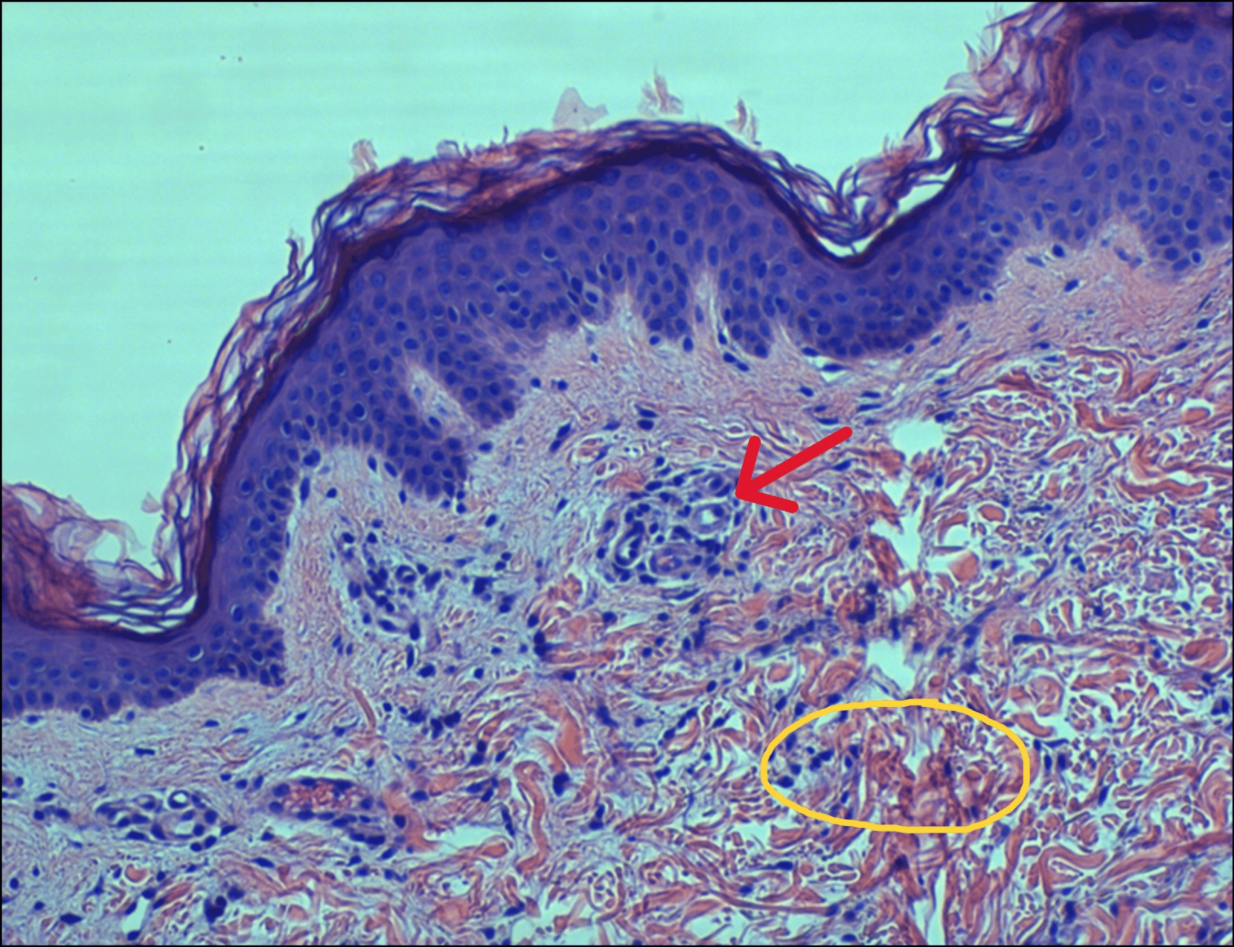
Hematoxylin and eosin (H&E) stain showing interstitial histiocytic infiltrates dissecting between collagen bundles with increased dermal mucin Red arrow: Histiocytic infiltrate dissecting collagen bundles in the dermis Yellow oval: Collagen bundles

**Figure 3:**
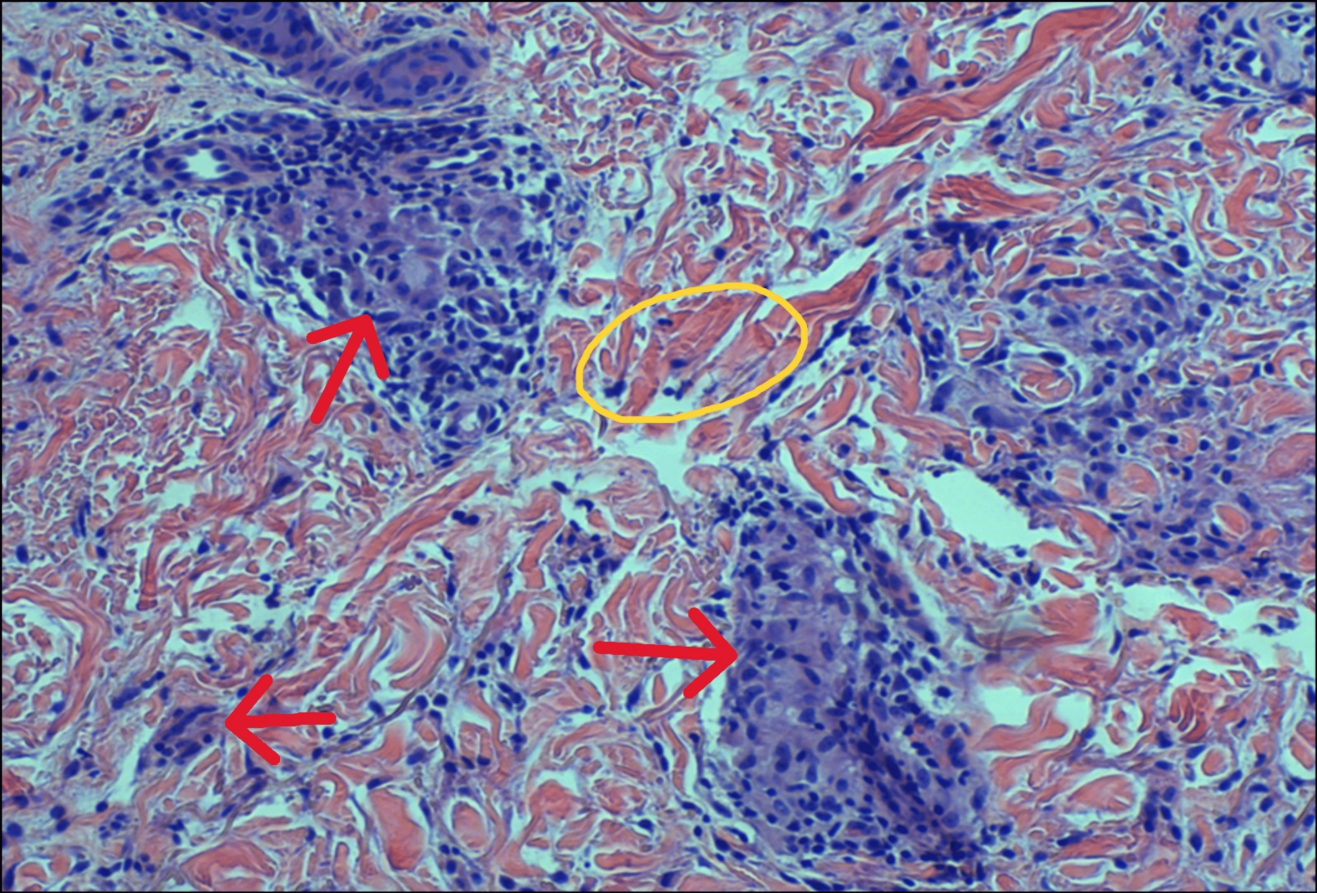
H&E stain at higher magnification Red arrows: Histiocytic infiltrates dissecting collagen bundles in the dermis Yellow oval: Collagen bundles

At follow-up five months later, the rash had not improved, prompting skin biopsy. A 4 mm punch biopsy of the left leg was performed, and histology revealed a focal increase in dermal mucin and histiocyte collections dissecting collagen bundles, findings most consistent with interstitial GA. No fungal organisms were identified on periodic acid-Schiff (PAS) stain, and there were no histological features suggestive of intertrigo, inverse psoriasis, Paget’s disease, or lichen sclerosus. Fite stain was negative. Biopsy results were discussed with the patient, and triamcinolone 0.1% ointment and hydroxychloroquine 200 mg twice daily for 90 days were initiated. The patient is currently pending follow-up to assess her response to treatment. Figures 2, 3 represent the histological findings of the left leg punch biopsy.

## Discussion

Diagnosing GA can be challenging due to its ability to mimic other skin diseases, including intertrigo, sarcoidosis, necrobiosis lipoidica, dermatomyositis, psoriasis, tinea cruris, mycobacterial infection, and several others. GA has also been reported as a granulomatous drug eruption with triggers such as TNF-α inhibitors, immune checkpoint inhibitors, monoclonal antibodies, and vaccines [1,2]. Dermoscopy can aid in diagnosis, and findings may reveal unfocused vessels of variable appearance against a pink-red background, but this is not pathognomonic [2]. Histologically, GA is often characterized by the triad of degraded collagen, histiocytic infiltration, and mucin deposits, which were all observed in our case. Abundant mucin is a hallmark of GA, which can be helpful in distinguishing GA from other similar dermatoses [2,5]. GA has been linked to diabetes mellitus, hyperlipidemia, and autoimmune conditions such as hypothyroidism, rheumatoid arthritis, and systemic lupus erythematosus. Our patient’s history of hypothyroidism, multiple sclerosis, and hyperlipidemia aligns with these associations, supporting the hypotheses that immune and metabolic dysfunction play a role in pathogenesis [2-6]. While our case’s histology findings and associated comorbidities are consistent with prior reports of GA, the localization to the inguinal folds is unusual and sparsely documented. This case therefore adds to the limited body of literature describing intertriginous GA, an anatomical variant that may be underrecognized due to its clinical resemblance to other dermatoses such as intertrigo or tinea.

A similar diagnostic challenge to our case is highlighted in a case report by Orleans et al., where GA localized to the bilateral groin was mistaken for tinea cruris and treated with antifungal therapy prior to histological confirmation [4]. In this particular case, the patient’s lesions eventually resolved spontaneously over three months without intervention, consistent with the typically self-limiting course of many GA cases [2,4,5].

In contrast, the outcome of our current case remains unknown as we await the patient’s response to hydroxychloroquine therapy. This therapy was initiated given the patient’s groin involvement, refractory disease, and impact on quality of life. Published reports describe partial and complete responses of GA to hydroxychloroquine; its favorable safety profile and immunomodulatory action support use in special-site disease or after failure of local therapy [2,7].

Both cases underscore the potential for GA to masquerade as more common conditions in the intertriginous region, an anatomical distribution of GA that is atypical with limited documentation in literature. Given its ability to mimic other dermatoses, granuloma annulare presents a diagnostic challenge. Prompt identification and management are essential to minimizing morbidity and enhancing quality of life.

## Conclusions

Granuloma annulare can be diagnostically ambiguous as it may clinically mimic a wide range of inflammatory, infectious, and iatrogenic dermatoses. Maintaining a broad differential is crucial, especially for lesions that are refractory to standard treatments. Our case underscores the novelty of GA localized to the bilateral inguinal folds, an atypical anatomic site with few documented reports. Histopathologic confirmation was critical in establishing the diagnosis, and the patient’s comorbidities of hypothyroidism, multiple sclerosis, and hyperlipidemia align with systemic associations frequently reported in GA. While spontaneous resolution can occur, chronic or treatment-resistant cases of GA require an individualized therapeutic approach; in this case, hydroxychloroquine was initiated with follow-up pending. By presenting this atypical case, our report contributes to the limited literature on intertriginous GA and underscores the importance of biopsy in refractory groin lesions.
